# Contraception in HIV-positive female adolescents

**DOI:** 10.1186/1742-6405-8-19

**Published:** 2011-06-01

**Authors:** Nadia T Kancheva Landolt, Sudrak Lakhonphon, Jintanat Ananworanich

**Affiliations:** 1 The HIV Netherlands Australia Thailand Research Collaboration (HIV-NAT) and The Thai Red Cross AIDS Research Center, Bangkok, Thailand; 2 SEARCH, Bangkok, Thailand; 3 Faculty of Medicine, Chulalongkorn University, Bangkok, Thailand

## Abstract

Sexual behavior of HIV-positive youths, whether infected perinatally, through risky behavior or other ways, is not substantially different from that of HIV-uninfected peers. Because of highly active antiretroviral therapy, increasing number of children, infected perinatally, are surviving into adolescence and are becoming sexually active and need reproductive health services. The objective of this article is to review the methods of contraception appropriate for HIV-positive adolescents with a special focus on hormonal contraceptives. Delaying the start of sexual life and the use of two methods thereafter, one of which is the male condom and the other a highly effective contraceptive method such as hormonal contraception or an intrauterine device, is currently the most effective option for those who desire simultaneous protection from both pregnancy and sexually transmitted diseases. Health care providers should be aware of the possible pharmacokinetic interactions between hormonal contraception and antiretrovirals. There is an urgent need for more information regarding metabolic outcomes of hormonal contraceptives, especially the effect of injectable progestins on bone metabolism, in HIV-positive adolescent girls.

## Introduction

Nong is just 15 years old. She was born in a remote part of Thailand, infected with HIV since birth. Her father died when she was one year old and her mother passed away when she was six. Nong had a sister, but she also died many years ago from AIDS. From an early age, Nong was cared for by an old man, a distant relative whom she calls "grandfather".

Nong is smart and very sensitive. She likes going to school, she is interested in drama and likes Thai boxing. She does not like to talk to adults, and prefers chatting with her teenage friends.

He was a boy from her school. They became friends. When "it" happened she was scared and did not know what to do. When Nong came to our clinic in January 2010 she was six months pregnant. She considered termination, but the pregnancy was too advanced. She cried continuously and said that she wished she was back at school. She did not want to stay in her own community while pregnant. Nong moved to a temporary government home for children and youths. She had good ARV adherence and was determined for her baby not to be born with HIV. In May she gave birth to a healthy little boy. A week after delivery she gave her baby to an orphanage. In June, she went back to school. On the last home visit she asked about her baby and said that she was happy at school.

## Background and objective

The objective of this article is to discuss methods of contraception appropriate for HIV-positive adolescents through a literature review, with special focus on hormonal contraception (HC).

According to the 2009 AIDS epidemic update, UNAIDS [1], there are still close to 400 000 HIV infected babies being born to HIV-positive mothers each year. Due to highly active antiretroviral therapy (HAART), an increasing number of children, infected perinatally, are surviving into adolescence. Adolescents with perinatally acquired HIV infections are becoming sexually active and are in need of reproductive health services [2]. Furthermore, approximately a quarter of all HIV-positive people, infected in various ways, are below 24 years of age. Half of this population is female.

The sexual behavior of HIV-positive youths is not substantially different from that of HIV-uninfected peers. They are young and inexperienced but curious, and sometimes under the influence of substances [3]. A number of studies looking into this topic have been conducted in the United States. One of them from 2001 included almost 400 girls between 13 and 19 years of age from the Reaching for Excellence in Adolescent Care and Health (REACH) cohort [4]. There were about 100 pregnancies over a period of three years. No significant difference in pregnancy incidence was detected between HIV infected and uninfected participants. However, the authors noted that among female adolescents who already had children at entry, HIV infected females were significantly less likely to become pregnant than HIV uninfected. The study comes to a conclusion that there is a need to design better contraceptive services and reproduction-related education targeting high-risk youth.

Another study with 156 HIV-positive adolescents (13 to 21 years old), infected perinatally or through risky behavior, also in the United States, showed that close to 2/3 of the participants were sexually experienced and approximately half of them had engaged in risky behavior since becoming aware of the diagnosis [5]. Forty-six of the 99 perinatally infected participants reported to have had sex. Nineteen out of 55 sexually active girls had been pregnant. The authors rightly conclude that the risky sexual behavior of perinatally infected adolescents is greater than previously estimated and points to the need of appropriate educational tools for early guidance in sexuality and health of these young people.

Similar trends are noted in other parts of the world. A study conducted in Bangkok, Thailand, that involved 70 HIV-positive young people between 16 and 25 years of age showed similar results regarding risky sexual behavior - almost half of the participants practised inconsistent condom use [6].

In Brazil between 2000 and 2008, 4900 HIV infected pregnant adolescents aged between 10 and 19 years were reported [7]. The authors found that the timing of first sexual intercourse and pregnancy among perinatally infected girls is comparable to what is observed in the general adolescent population. The Pan American Health Organization reported that in Latin America, 50% of youths below 17 years of age are sexually active and up to 25% of all babies are born to adolescents [8].

HAART gives children with HIV a chance to live, grow up and enjoy life, including sex. It is the role of health professionals to give young people the possibility to practise sex in a safe way. Appropriate sexual and contraceptive practice would help prevent the transmission of HIV and other sexually transmitted diseases (STDs) as well as unintended pregnancies [9]. It is increasingly important that HIV-positive adolescent girls are offered reliable family planning services.

### What contraceptive methods are suitable for HIV-positive adolescents?

A substantial choice of contraceptive methods exists - abstinence, barrier methods, natural methods, HC, intrauterine devices (IUD), sterilization and spermicides [9,10]. It is unrealistic to expect all young people to adhere to abstinence [8]. So far, a perfect method, which provides effective contraception and STDs prevention while having no side effects, and is completely accepted by users in all situations, does not exist. This is even more relevant in case of an STD such as HIV infection when prevention of STD/HIV transmission is restricted to using a barrier method that may not be acceptable to many users. The protective benefit of male condom is well documented [11]. But male condoms are all too often not used as recommended, especially by young people [12]. Therefore, in case of lack of abstinence, the use of two methods, one of which is the male condom, is currently the most effective option for those who desire simultaneous protection from both pregnancy and STDs [3,13,14]. Health workers who provide family planning and STD/HIV prevention services should continue to promote the dual protection method even if uptake can be low especially among young users [15].

A study published in March 2010 gave a population estimate that if all women in the United States who use one highly effective contraceptive method added a second one, such as the condom, then approximately 80% of unintended pregnancies and abortions among these women could be prevented. This would result in an annual reduction of 786,000 unintended pregnancies and nearly 152,000 abortions [16].

When advising a young girl on available contraceptive choices, we must take into consideration a number of factors such as her young age and inexperience, a dynamic pattern of life and sexual relationships, and of course the concomitant condition of HIV infection and ARV use. Advice on sexual risk and ways for delaying the start of sexual life should begin as early as possible before young people get used to risky behavior; ideally before their first sexual intercourse. Today many programs on sexual education to adolescents are developed and implemented on different levels - parents, schools, community - via professional educators or peers.

Though HIV-positive adolescents, and particularly those on HAART, constitute a particular group, at present there is not enough evidence related to the choice of contraceptive method for this group. Sometimes we have to give an empiric advice, based on information about contraceptive use in adolescents in general, or about contraceptives in HIV-positive women of any age.

Highly effective modern contraceptive methods include HC, IUD and sterilization (male and female). These methods do not protect from STD/HIV, but their contraceptive effect is very high (< 0, 5 pregnancies/100 women/year in case of perfect use of the method).

**Sterilization **has many advantages as it provides excellent prevention of pregnancy and does not have the side effects of other contraceptive methods and has no pill burden. The disadvantage is that sterilization is a virtually irreversible choice and its use raises serious ethical questions, especially if it is conducted without adequate counseling or if HIV-positive women are forced to undergo the procedure as reported in some countries [17]. In addition it requires an operative procedure.

Although sterilization might be a choice for older HIV-positive women who no longer desire to have children, it is not a plausible option for adolescent girls. A recent study in the general population showed that women who undergo sterilization at a young age may later regret this decision [18]. In this study women who underwent sterilization at the age of 30 years or younger were twice as likely as those over 30 to express regret. They were also 3.5 to 18 times more likely to request information about reversing the procedure and about 8 times more likely to undergo sterilization reversal or an evaluation for in vitro fertilization.

**The IUD**, containing either copper or progestin, is the most popular reversible long acting contraceptive method used in the world. As compared to HC, the IUD has the advantage of lacking pill burden, the need for regular application and the adverse events associated with hormonal components in the HC methods. The progestin-releasing IUD has an advantage over the copper IUD in reducing menstrual bleeding. A study conducted in Zambia in 2007 reported that the IUD is a safe and effective method of contraception in HIV-positive women [19]. The study randomly assigned 303 women to HC and 296 women to copper IUD. Women who were assigned to HC were more likely to become pregnant than those who were assigned to IUD (4.6 vs. 2.0/100 woman-years). Only one woman who was assigned to the IUD group experienced pelvic inflammatory disease (crude rate, 0.16/100 woman-years), and there was no pelvic inflammatory disease among those women who were assigned to HC.

Earlier reports showed higher incidence of adverse events such as dysmenorrhea, expulsion, impaired restoration of fertility with prolonged use of IUD in nulliparous and young women [20,21]. One study assessing the use of Copper IUD (Copper T380) in 39 adolescent mothers [22], found that six users had partial or complete expulsion (15%), and 10 requested removals (26%) within 24 months of placement. Furthermore, four users (10%) became pregnant - three had an IUD in place at time of conception, while one became pregnant due to unrecognized device expulsion. The authors noted that even though many adolescent mothers discontinued IUD use within two years of placement, the numbers of patients were too small to provide stable estimates of contraceptive effectiveness. The study supports the need for further studies of IUD in adolescents.

On the other hand more recent studies find that IUD is a safe and effective long-term contraceptive method for the above discussed population [23]. The article concludes that because adolescents contribute disproportionately to the epidemic of unintended pregnancy, IUDs should be considered as a first-line contraceptive choice regardless of parity. The World Health Organization (WHO) puts the IUD for nulliparous and women below 20 years of age in category 2 - the advantage of using the method generally outweighs the theoretical or proven risk [24].

### HC for HIV-positive adolescents - questions to be answered

HC is a highly effective modern method of contraception. There are two main types of HC. The first one is the combined estrogen and progestin type which includes the combined oral contraceptive pill (COC), the skin patch or the vaginal ring. The second one is the progestin-only type which could be delivered as a pill, a depot injection or an implant.

Despite the topic of HC being discussed in recent years in several excellent review articles [25-28], there are few original studies in this field. When considering HC for HIV-positive adolescents, one should think about the following issues: HIV disease progression, genital tract HIV shedding and infectivity, pharmacokinetic (PK) interactions between hormones and ARVs and last, but not least - metabolic outcomes.

### HIV Disease progression

Data on the effect of HC on HIV disease progression is still inconclusive. Sex steroid hormones influence the immune system; progesterone can have a suppressive effect whereas estrogens can have the reverse [29,30]. The exact mechanisms are not clearly understood. In addition to influence on the immune system, estrogens and progesterone have an effect on the structure of the vaginal epithelial wall and the vaginal microorganisms. Epidemiologic studies in humans [31,32] and challenge studies in ovariectomized macaques [33,34] suggest that progesterone-based contraceptives increase the transmission risk of HIV-1 infection in humans and of simian immunodeficiency virus (SIV) infection in macaques due to increase viral shedding in the genital tract, while estrogens made macaques resistant to SIV, mainly due thickening of the vaginal epithelium.

Baeten et al. demonstrated in the Mombasa cohort that the use of depot medroxyprogesteron acetate (DMPA) at the time of HIV infection was associated with a higher plasma HIV-1 viral load set point, which predicted faster progression of the HIV-1 disease [31]. They also showed that women using HC, COC or DMPA, at the time of HIV infection were more likely to acquire multiple HIV viral genotypes, which in turn was associated with higher HIV plasma viral load set point and faster CD4 T cell decline. A study conducted in Zambia suggests that HC might enhance disease progression if administered in HIV-positive women prior to ARV [32]. The researchers randomized 599 HIV-infected women to either a non-hormonal contraceptive method, such as an IUD, or a HC, such as COC pill or DMPA. Disease progression was defined either as death or becoming eligible for ARV. They found that COC or DMPA use was associated with HIV disease progression among women not yet on ARV.

On the other hand, data from a multi-country cohort analysis involving 4,000 women, published at the end of 2009 by the same group of researchers, did not find an effect of exogenously administered progesterone on HIV-1 acquisition and disease progression [35].

There are several other studies published in recent years which confirm the same observation. A study from Uganda with 625 women finds that HC is not associated with progression to death and is actually associated with reduced progression to AIDS [36]. In HIV-infected postpartum Kenyan women, the results were similar with no significant immediate or longer-term effects of the use of COC or DMPA on HIV-1 plasma viral load and CD4 T-cell counts [37]. The role of HC in the effectiveness of HAART was examined among participants in the Women's Interagency HIV Study who were followed from HAART initiation [38]. The authors did not find any substantial evidence that use of HC strongly affected responses to HAART.

Scientific evidence is currently not conclusive about HIV progression and contraceptive use. HC use in women on HAART does not show progression and there are not enough studies in HIV infected adolescents in this regard.

### PK interactions between hormones and ARVs

Another important consideration involving HC in HIV positive women is the PK interaction between hormones and ARV drugs. There are several review articles [26-28] on the topic and only a limited number of studies with mostly small sample size and short duration of exposure to both hormones and ARVs. Most of the studies are based on the assumption that as sex steroid hormones are predominantly metabolized via the cytochrome P450 system. Any medication which affects this pathway, such as some ARVs would change the PK of estrogens and progestins. The decrease in hormone levels could potentially decrease the contraceptive effect whereas the increase in hormone levels could potentially increase hormone-related side effects (e.g. thromboembolism). However, the area under the curve (AUC) and the maximal concentration (Cmax) of any drug are dependent on multiple factors such as age, body weight, hormonal cycles of exposure to the drug (HC, ARVs), the specific drug molecule and its dosage. Furthermore, in adolescents, activity of drug metabolizing enzymes is influenced by the physical and sexual development, with greatest variability in puberty [39]. However, information in this field is limited and there is need for further studies. Last, but not least, PK differences of sex steroid hormones may not be as important as the direct indicators of pregnancy risk such as ovulation during the oral contraceptive cycle [40-42].

Nevertheless, we will briefly present the studies conducted in this field. We will divide the PK studies into two groups depending on the HC: progestin-only HC (DMPA) and combined, estrogen and progestin hormonal contraceptive.

### PK studies of DMPA and ARVs

There are two studies assessing the interaction between DMPA and ARVs (Table 1). Cohn, Watts et al. [43,44] enrolled 70 HIV-positive women, 22 to 46 years of age, in four groups depending on their ARV regime - nucleoside reverse transcriptase inhibitors (NRTI) only, two NRTIs and one protease inhibitor (PI), nelfinavir (NFV), two NRTIs and one non-nucleoside reverse transcriptase inhibitor (NNRTI), either efavirenz (EFV) or nevirapine (NVP). The NRTI-only group served as a control group, as NRTIs have a different metabolic pathway and are not expected to have an impact on PKs of sex hormones. DMPA was administered once during the study in a standard dose of 150 mg by intramuscular injection. Blood levels of DMPA, progesterone and respectively of ARVs, NFV, NVP and EFV were measured. There were no significant changes in medroxyprogesterone acetate levels in any of the three groups (NFV, EFV or NVP) compared to the control group. Minor changes in NFV and NVP drug exposure were seen after DMPA, but were not considered clinically significant. Suppression of ovulation, assessed by progesterone levels, was maintained. The study concludes that DMPA can be used safely by HIV-positive women on the ARVs studied.

**Table 1:** PK studies assessing the interaction between HC and ARVs

PK studies assessing the interaction between DMPA and ARVs					
	**Author**	**n**	**ARV**	**Hormones**	**Outcome**

1.	Cohn, Watts et al, 2006 (43, 44), pharma sponsored partly	70 HIV+, 22-46 y	NFV (n = 21) EFV (n = 17) NVP (n = 16) NRTI only (n = 16)	DMPA, single dose	↓ NFV ↑ NVP EFV - no significant change DMPA - no significant change Progesterone < 1.6 ng/ml, no ovulation Conclusion: DMPA is an effective contraceptive method for HIV+ women on ARVs in the study

2.	Nanda et al., 2007 (45)	30 HIV+, 19-40 y	AZT+3TC+EFV	DMPA, single dose	ARV levels - not done DMPA - no significant change Progesterone - only in one woman (control group) > 5 ng/ml, might indicate ovulation Conclusion: DMPA is an effective contraceptive method for HIV+ women on triple ARV regime in the study

**PK studies assessing the interaction between EE, progestins and NNRTIs and NtNRTI**					

1.	Mildvan et al., 2002 (46), pharma sponsored	10 HIV+, 26-47 y	NVP 200 mg BID	0.035 mg EE/1.0 mg NET, single dose	↓ 29% AUC of EE ↓ 18% AUC of NET NVP - no significant change Conclusion: COC should not be primary method for contraception in HIV+ women on NVP

2.	Joshi et al., 1998 (47), pharma sponsored	13 HIV-	EFV 400 mg OD, 7 days	0.05 mg EE, single dose	↑ 37% AUC of EE EFV - no significant change Conclusion: no decrease in EE levels when co-administered with EFV

3.	Sevinsky et al., 2008 (48), pharma sponsored	28 HIV-, 18-42 y	EFV 600 mg OD, 14 days	EE/NGM, 3 cycles	EE - no significant change ↓ 64% AUC of NGMN ↓ 83% AUC of LNG EFV - no significant change Progesterone < 1.25 ng/ml Conclusion: need of reliable barrier contraception when taking COC with EFV

4.	Scholler-Guyera et al., 2009 (49), pharma sponsored	30 HIV-, 18-45 y	ETR 200 mg BID	0.035 mg EE/1.0 mg NET, 3 cycles	↑ 22% AUC of EE ↓ 5% AUC of NET ↑ ETR Conclusion: no compromise in contraceptive effect

5.	Kearney et al., 2009 (50), pharma sponsored	20 HIV-, 19-45 y	TDF 300 mg OD	EE/NGM, 3 cycles	EE - no significant change NGM - no significant change TDF - no significant change Conclusion: TDF does not alter PK of EE and NGM

**PK studies assessing the interaction between EE, progestins and PIs**					

1.	Ouellet et al., 1998(51), pharma sponsored	23 HIV-, 18-45 y	Ritonavir 500 mg BID	0.05 mg EE, single dose	↓41% AUC of EE Conclusion: use an alternative contraceptive method when ritonavir is administered

2.	Frohlich et al., 2004(54), pharma sponsored partly	8 HIV-, 23.8 y	Saquinavir single dose	0.03 mg EE 0.075 mg gestoden	SQV - no significant change Conclusion: COC does not alter single dose saquinavir

3.	Tacket et al., 2003 (55), pharma sponsored	22 HIV-	ATZ 400 mg	0.035 mg EE/1.0 mg NET	↑ 48% AUC of EE ↑110% AUC of NET Conclusion: no compromise in contraceptive effect, no dose adjustment needed

4.	Sekar et al., 2008 (52), pharma sponsored	19 HIV-	DRV/r 600 mg/100 mg BID	0.035 mg EE/1.0 mg NET, 2 cycles	↓44% AUC of EE ↓14% AUC of NET Conclusion: use an alternative method

5.	Vogler et al., 2010 (53)	8 HIV+ with LPV/r, 24 HIV+ w/o LPV/r	LPV/r 400 mg/100 mg	0.035 mg EE/1.0 mg NET, single dose EE/NGMN skin patch for 3 w	↓45% AUC of patch EE ↑83% AUC of patch NGNM ↓55% AUC of pill EE ↓19% AUC of LPV with patch ↓23% AUC of RTV with patch Progesterone < 2.88 ng/ml, no ovulation Conclusion: PK of EE/NGMN significantly altered, but clinical effect probably not affected

NFV = nelfinavir, EFV = efavirenz, NVP = nevirapine, NRTI = nucleoside reverse transcriptase inhibitor, AZT = zidovudine, 3TC = lamivudine, DMPA = depot medroxyprogesterone acetate, EE = ethinyl estradiol, NET = norethindrone, NGM = norgestimate, NGMN = norelgestromin, LNG = levonorgestrel, ETR = etravirine, TDF = tenofovir disoproxil fumarate, RTV = ritonavir, ATZ = atazanavir, SQV = saquinavir, DRV/r = ritonavir boosted darunavir, LPV/r = ritonavir boosted lopinavir, COC = combined oral contraceptive, PK = pharmacokinetic, AUC = area under the curve, ↑ = increase, ↓decrease, OD = once daily, BID = twice daily, pharma sponsored = the study is financed by a pharmaceutical company (manufacturer of drugs under study)

In 2007 Nanda et al. made similar findings in a group of women on two NRTIs and EFV [45]. A single standard dose of DMPA was administered. PK of DMPA was similar compared to a group of HIV-positive women without ARV. The authors conclude that the DMPA levels are not affected by EFV.

### PK studies of COC, ethinyl estradiol (EE)/progestin, and ARVs (NNRTIs, Nucleotide reverse transcriptase inhibitors (NtRTI) and PIs)

NRTIs include molecules such as zidovudine (AZT), stavudine (d4T), lamivudine (3TC), didanosine (ddI), abacavir (ABC) and others. NRTIs are not metabolized via the cytochrome P450 system; therefore, they are not expected to influence sex steroid hormone levels. The same is valid for the NtRTI, among which the drug tenofovir (TDF). At present, NRTIs are the backbone of standard ARV regimes.

The NNRTIs, such as NVP and EFV are part of most first line therapies. Etravirine (ETR) is a second generation NNRTI that has higher threshold for resistance compared to EFV and NVP. They are metabolized primarily via the cytochrome P450 path; therefore, interactions with sex steroid hormones are expected in both directions. The studies evaluating the interaction between EE, progestin and NNRTI or NtRTI are listed in Table 1.

The interaction between NVP and EE with norethindrone (NET) was studied by Mildvan et al. in 10 HIV-positive women [46]. The selected participants had to be on a stable triple ARV regime for at least one month prior to enrollment in the study. The ARV regime did not have NVP or ritonavir (RTV). On day 0 a single dose of 0.035 mg EE/1.0 mg NET was given, followed by measurement of EE and NET levels. NVP was added to the therapy in an initial dose of 200 mg once daily for 15 days, followed by 200 mg twice daily for additional 15 days. On day 30 another single dose of 0.035 mg EE/1.0 mg NET was given and blood levels of EE, NET and NVP were measured. The authors found a 29% reduction of EE and an 18% reduction of NET levels in AUC. NVP levels were not changed significantly compared to historical data. They concluded that COC should not be the primary method of birth control in women of child-bearing potential who are treated with NVP.

There are two small studies assessing the interaction between EFV and EE or EE/NGM (norgestimate) containing COC, and the active metabolites of NGM - norelgestromin (NGMN) and levonorgestrel (LNG) [47,48]. They were conducted in a small sample of HIV-negative women, the first one with 400 mg EFV for one week and 0.050 mg EE only, and the second one with 600 mg EFV for two weeks prior to EE/NGM administration. The first study found increase in EE levels and no significant changes in EFV levels [47]. The other also found no change in EFV levels, as well as no change in EE levels [48]. The progestin levels, NGMN and LNG, were significantly reduced; though participants most probably did not have ovulation (serum progesterone remained low). In conclusion there is a need to use reliable barrier contraception when taking COC with EFV.

The effect of ETR on PK of 0.035 mg EE/1.0 mg NET was assessed in 30 HIV-negative women [49]. The authors concluded that the changes in EE and NET were not clinically relevant, and no loss in contraceptive efficacy was expected when hormones were co-administered with ETR.

The potential interaction between EE/NGM was assessed with TDF [50]. PK parameters for NGM and EE were unaltered by co-administration of TDF, as were the levels of TDF.

There are published studies on PK interaction between several PIs and COCs (Table 1). In 1998 Ouellet et al. [51] administered RTV, as a single PI in healthy volunteers, in gradually increasing doses for 30 days from 300 mg to 500 mg twice daily. At the same time on day 1 and day 29, 0.05 mg of EE was administered. The EE blood levels measured on day 29 were reduced by approximately 40% in comparison to day 1. The authors consider the reduction significant and recommend the use of an alternative contraceptive method when RTV is used. In clinical practice RTV is primarily used in lower doses, usually 100 mg twice daily, as a booster to PIs. We found two studies assessing PK interaction between EE and RTV boosted PIs [52,53]. Both studies found reduction in the EE levels when co-administered with RTV boosted PI, which the authors considered significant. We present details of these studies further in the article.

Frohlich et al. [54] studied the effect of 0.03 mg EE/0.075 mg gestodene on saquinavir (SQV) in healthy volunteers based on the premise that women had more adverse drug reactions to ARVs than men. The results showed no effect of OC on SQV PK.

There are two small studies in HIV-negative women conducted by a manufacturer with atazanavir (ATV) [55] and low dose RTV-boosted darunavir (DRV/r) [52]. The administration of 400 mg of ATV without boosted RTV, with 0.035 mg EE/1.0 mg NET led to a 48% and 110% increase of AUC of EE and NET, respectively. No dose adjustment of COC was recommended. In the second study of DRV/r, there was a decrease of 44% in EE AUC and 14% in NET AUC, respectively. The PK interaction observed here is considered to be clinically significant and the study recommends the use of an alternative or additional contraceptive method.

The interaction between Maraviroc, a CCR5 receptor agonist and 0.03 mg EE/0.15 mg LNG in 14 HIV-negative women was assessed [56]. The concentrations of EE and LNG remained similar with and without Maraviroc. There are several other studies that assessed the interactions between indinavir (IDV), amprenavir (APV), nelfinavir (NFV), and COC in HIV-negative women [57-59]. After co-administration of 800 mg IDV three times daily with 0.035 mg EE/1.0 mg NET, slight increase of AUC of EE (22%) and NET (26%) was observed. PK assessment of APV with EE/NET showed similar results - no significant change in EE levels and slight increase in NET AUC of 18%. The contraceptive effect of COC is not compromised when administered with IDV and APV. NFV (750 mg) administered three times daily with 0.035 mg EE/1.0 mg NET led to 47% decrease of AUC of EE and 18% of NET, which might require the use of an additional or alternative method of contraception.

One interesting study by Vogler et al. reported the interaction between RTV-boosted lopinavir (LPV/r) and EE/NGMN delivered through a skin patch in HIV-positive women [53]. There were eight participants with HC and triple ARV regime including LPV/r 400 mg/100 mg twice daily and two NRTIs, and a control group without ARV, or NRTI only regime, of 24 participants. After a single dose of COC (0.035 mg EE/1.0 mg NET), EE and NET levels were measured. Two days later a patch was administered for three weeks, changing every week. EE and NGMN levels were measured multiple times during the study period. The AUC of EE was decreased in the LPV/r group compared to the control group for both the COC (55%) and the patch (45%), while the AUC of NGMN was increased by 83% in the LPV/r group in comparison to the control group. The AUC of LPV was decreased by 19% and of RTV by 24%, respectively. Serum progesterone levels remained low in all participants, showing no signs of ovulation. The authors concluded that although PKs of contraceptive EE and NGMN were significantly altered with LPV/r, the contraceptive efficacy of the patch was likely maintained.

All existing data point to the conclusion that despite a decrease or increase in the blood levels of sex steroid hormones, the efficacy regarding their contraceptive effect is most probably not compromised, as all major studies measured also levels of progesterone at least once [43-45,48,53]. Serum progesterone levels remained always below 5 ng/ml, values consistent with anovulation [60]. Only in one woman serum progesterone was above 5 ng/ml [45]. She was from the control group, without ARV, and the HC under study was DMPA, with which suppression of ovulation is not the only way of ensuring contraceptive effect. Despite this outcome, in all major guidelines [10], the recommendation is to use an alternative or additional method of contraception when using combined HC with NNRTIs (NVP or EFV), or RTV-boosted PIs. All of these studies, with the exception of two [45,53], were conducted by the research department of the pharmaceutical companies [46-52,55-59], or partly co-funded by the pharmaceutical companies [43,44,54]. More studies with higher numbers of participants in "real-life situations" are needed to confirm this conclusion.

We did not find studies evaluating to what extent this increase or decrease in blood levels of EE and progestin affects the non-contraceptive effects of HC. Some of these effects, such as improvement of acne and reduction of menstrual pain could be very beneficial, especially among adolescent girls [61]. Others, such as deep venous thrombosis, might be fatal and there is an urgent need for more information in this field.

### Metabolic and bone outcomes of hormonal contraceptives in female adolescents and adults

Because of the potential effects of HIV infection, ARV and hormones themselves on body metabolism, we might expect more changes in plasma lipids and glucose tolerance in HIV-positive women using HC, especially with progestin-only HC. The issue has been studied by Womack et al [62] who enrolled HIV-infected and uninfected women in the Women's Interagency HIV Study (WIHS), an ongoing multicenter longitudinal cohort study of the progression of HIV infection in women. The authors found that progestin-only and combined HC impact metabolic outcomes differently. Progestin-only HC was associated with lower high density lipoprotein (HDL) and greater insulin resistance in HIV-infected and uninfected women. On the other hand, combined HC was associated with higher HDL in HIV-infected and uninfected women. This information is of particular interest as progestin-only HC is gaining popularity among HIV-positive women due to the clearer pattern of PK interaction with ARVs.

The impact of HC on bone density in HIV-positive adolescents is unfortunately much less studied. HC, especially DMPA and to a lesser extent low dose COC, has been associated with loss of bone mineral density (BMD) in adolescents [63], regardless of their HIV status.

In 2004, the US Food and Drugs Administration (FDA) added a black box warning to the package insert of DMPA [64], stating that women who use DMPA contraceptive injection may have significant BMD loss and that it is unknown if the use of the DMPA during adolescence or early adulthood, a critical period of bone accretion, will reduce peak bone mass and increase the risk of osteoporotic fracture later in life. They recommend not using this method for more than two years, because of a possible decrease in the amount of calcium in the bones, especially in case of an additional factor such as smoking or drinking which carries a risk of osteoporosis.

This FDA black box provoked much discussion and many studies were conducted in this field. A multicentre study in the USA with 98 long term DMPA users (up to 240 weeks) between the ages of 12 to 18 years concluded that BMD loss in female adolescents receiving DMPA is substantially or fully reversible in most girls following discontinuation of DMPA, with faster recovery at the spine level than at the hip [65].

A study conducted in Thailand [66] on the long-term use of DMPA on BMD found that long-term use of DMPA had a negative impact on lumbar spine BMD.

We did not identify similar studies conducted in HIV-infected adolescents or women. There is an urgent need to study the topic in this group as DMPA is considered safe to use with HIV infection due to its favorable interaction with ARVs [43-45]. On the other hand, low BMD is prevalent in HIV infected women [67,68]. The start of HAART leads to a 2% to 6% decrease in BMD over the first 2 years, due to multiple factors, among which are HIV infection, ARVs, traditional osteoporosis risk factors, and increased fracture rates in the HIV-infected population. Mora et al. studied the growth of skeletons in children and adolescents, and found a typically high bone cell activity [69]. The study found that HAART-treated children had higher levels of bone formation and bone resorption compared with healthy controls, with an association between ARV and enhancement of bone metabolic rate. An increased rate of bone turnover causes BMD decrease. The authors also found a relation between the severity of osteopenia and lipodystrophy.

After reviewing available data on HC in HIV-infected women and finding practically no information on HIV-infected adolescent girls, we consider that the PK interaction between HC and ARVs is perhaps of higher importance in its impact on metabolism and bone than on contraception in this group.

### Emergency contraception (EC)

At the end of this review article we assess possible options for EC in HIV-positive adolescents. This is not a form of contraception to be used on a regular basis and should be kept only for emergency situations, such as condom breakage.

A study in Thailand assessed the proportion of adolescent mothers aged 19 years or less, regardless of their HIV status, who were aware of EC [70]. The study was conducted with 104 girls at antenatal or postnatal clinics and found that close to 85% of the adolescent mothers were aware of EC, though less than 30% had used it in the past. The authors summarize that health care providers should be the sources of information on contraceptive methods for adolescents.

EC can be achieved either by taking a progestin pill (1.5 mg of LNG) as soon as possible and not later than 72 hours after unprotected intercourse, or by inserting an IUD up to 5 days from unprotected sexual intercourse (before the possible implantation of a fertilized egg occurs). According to the 2008 British HIV Association Guidelines for the management of sexual and reproductive health of people living with HIV infection, quoting a statement of the Faculty of Family Planning and Reproductive Health Care, London [10], the dose of hormonal EC should be doubled in woman who is taking HAART. At the time of this recommendation there were no studies confirming that this dose increase was required. The guideline suggests that perhaps the IUD is a more appropriate method for EC in woman who is HIV-positive and on HAART.

Carten et al. reported the results of the first prospective study assessing the effect of EFV on PK of 0.75 mg LNG, used as a hormonal EC [71]. EFV reduced the AUC of LNG significantly (> 50%). The authors confirmed the recommendations in the British HIV Association Guidelines, to increase the hormonal dose though they recognized that the minimum effective LNG concentration is not known. They also pointed out the importance of dual methods of contraception.

EC could be an important option of contraception in HIV-positive girls, who are well trained to use condoms, and will recognize a problem such as damage for instance. It has less pill and chemical burden on the body, though the effectiveness of a standard dose with concomitant ARV therapy is still unconfirmed.

## Conclusion

There are no studies at present assessing the contraceptive choice in adolescent HIV-positive girls. This is an emerging group of young people with HIV who are showing similar sexual risk behaviors as their non-HIV infected peers. The best contraceptive option is the one that works for the patient. Currently the most effective option for preventing STIs and unintended pregnancy is the dual method of male condom for STD/HIV prevention and either HC or IUD for pregnancy prevention. There is an urgent need to obtain more information regarding metabolic outcomes of HC, especially the effect of injectable progestins on bone metabolism, in adolescent HIV-positive girls. Health professionals should discuss sexual risk and contraceptive choices with HIV-positive adolescents as early as possible and preferably before the start of sexual life. The informed decision should be made by the adolescent girl based on her lifestyle, sexual activity and reproductive history.

Perhaps Nong could have been given a different choice...

## List of abbreviations

ABC: abacavir; APV: amprenavir; ARV: antiretroviral; ATZ: atazanavir; AUC: area under the curve; AZT: zidovudine; BID: twice daily; BMD: bone mineral density; COC: combined oral contraceptive; DMPA: depot medroxyprogesterone acetate; ddI: didanozine; D4T: stavudine; DRV/r: ritonavir boosted darunavir; EC: emergency contraception; EE: ethinyl estradiol; EFV: efavirenz; ETR: etravirine; FDA: US Food and Drugs Administration; HAART: highly active antiretroviral therapy; HC: hormonal contraception; HDL: high density lipoproteins; IUD: intrauterine device; IDV: Indinavir; LNG: levonorgestrel; LPV/r: ritonavir boosted lopinavir; NET: norethindrone; NFV: nelfinavir; NGM: norgestimate; NGMN: norelgestromin; NNRTI: non-nucleoside reverse transcriptase inhibitor; NtRT: nucleotide reverse transcriptase inhibitor; NRTI: nucleoside reverse transcriptase inhibitor; NVP: nevirapine; OD: once daily; PI: protease inhibitor; PK: pharmacokinetic; RTV: ritonavir; STDs: sexually transmitted diseases; SQV: saquinavir; TDF: tenofovir disoproxil fumarate; 3TC: lamivudine; ↑: increase; ↓decrease

## Competing interests

The authors declare that they have no competing interests.

## Authors' contributions

NKL reviewed the literature and drafted the manuscript. SL introduced the personal story of Nong. JA gave scientific input and edited the manuscript. All authors read and approved the final manuscript.
